# Diffusive Silicon Nanopore Membranes for Hemodialysis Applications

**DOI:** 10.1371/journal.pone.0159526

**Published:** 2016-07-20

**Authors:** Steven Kim, Benjamin Feinberg, Rishi Kant, Benjamin Chui, Ken Goldman, Jaehyun Park, Willieford Moses, Charles Blaha, Zohora Iqbal, Clarence Chow, Nathan Wright, William H. Fissell, Andrew Zydney, Shuvo Roy

**Affiliations:** 1 Division of Nephrology, University of California San Francisco, San Francisco, California, United States of America; 2 Department of Bioengineering and Therapeutic Sciences, University of California San Francisco, San Francisco, California, United States of America; 3 Ben Chui Consulting, Sunnyvale, California, United States of America; 4 H-Cubed, Olmsted Falls, Ohio, United States of America; 5 Department of Surgery, University of California San Francisco, San Francisco, California, United States of America; 6 Silicon Kidney, LLC, San Francisco, California, United States of America; 7 Division of Nephrology & Hypertension, Vanderbilt University Medical Center, Nashville, Tennessee, United States of America; 8 Department of Chemical Engineering, Pennsylvania State University, State College, Pennsylvania, United States of America; University Medical Center Utrecht, NETHERLANDS

## Abstract

Hemodialysis using hollow-fiber membranes provides life-sustaining treatment for nearly 2 million patients worldwide with end stage renal disease (ESRD). However, patients on hemodialysis have worse long-term outcomes compared to kidney transplant or other chronic illnesses. Additionally, the underlying membrane technology of polymer hollow-fiber membranes has not fundamentally changed in over four decades. Therefore, we have proposed a fundamentally different approach using microelectromechanical systems (MEMS) fabrication techniques to create thin-flat sheets of silicon-based membranes for implantable or portable hemodialysis applications. The silicon nanopore membranes (SNM) have biomimetic slit-pore geometry and uniform pores size distribution that allow for exceptional permeability and selectivity. A quantitative diffusion model identified structural limits to diffusive solute transport and motivated a new microfabrication technique to create SNM with enhanced diffusive transport. We performed in vitro testing and extracorporeal testing in pigs on prototype membranes with an effective surface area of 2.52 cm^2^ and 2.02 cm^2^, respectively. The diffusive clearance was a two-fold improvement in with the new microfabrication technique and was consistent with our mathematical model. These results establish the feasibility of using SNM for hemodialysis applications with additional scale-up.

## Introduction

End Stage Renal Disease (ESRD) remains a major public health problem in the United States. Over 692,000 Americans [1] suffer from ESRD as of 2014. ESRD imposes a disproportionately high financial burden compared to other chronic diseases with a total health care expenditure topping $49 billion in 2011 [1]. Kidney transplantation currently offers the best treatment option based on mortality rates, quality of life, and total cost [1–3]. However, scarcity of donor organs remains a major limitation to widespread transplantation [4]. As a result, the majority of ESRD patients in the United States undergo dialysis treatment with over 90% receiving in-center, 3–4 hour, thrice weekly hemodialysis. This remains the standard of care despite mounting evidence that longer and more frequent hemodialysis offers beneficial clinical outcomes [5–8]. Therefore, several groups, including ours, are attempting to shift the current renal replacement paradigm by developing technologies that would provide a portable or implantable form of renal replacement for patients [9–12].

Current high-flux hemodialyzer membranes, typically constructed from polysulfone or polyethersulfone, have wide pore-size distribution and long tortuous pore geometries that limit selectivity and permeability [13]. The hollow fiber configuration does allow for efficient use of surface area (1.5–2.5 m^2^ with small extracorporeal volume), but also results in a large pressure drop and bulky package size that does not easily allow for miniaturization [14]. Additionally, exposure to polymer membranes has detrimental effects on platelet function, increased oxidative stress, inflammation, and biofouling [15–18]. These problems are even more apparent when considering more frequent and longer dialysis treatment times. Consequently, conventional polymer hemodialysis membranes are likely limited in their capacity to provide an implantable or portable device that requires long-term continuous blood exposure.

In contrast, microelectromechanical systems (MEMS) technology allows for fabrication of membranes with reproducible and uniform pore size distribution [19,20] with tunable pore geometry that can be optimized for molecular transport and permeability [19]. Microfabricated silicon membranes also enable surface chemistry modification that can limit immunologic reaction and protein fouling, while enhancing selectivity based on electrostatic charge [21–24]. Furthermore, MEMS enables device miniaturization not feasible with current polymer hemodialyzers given their inherently large package size and high internal flow resistance.

**Fig 1:**
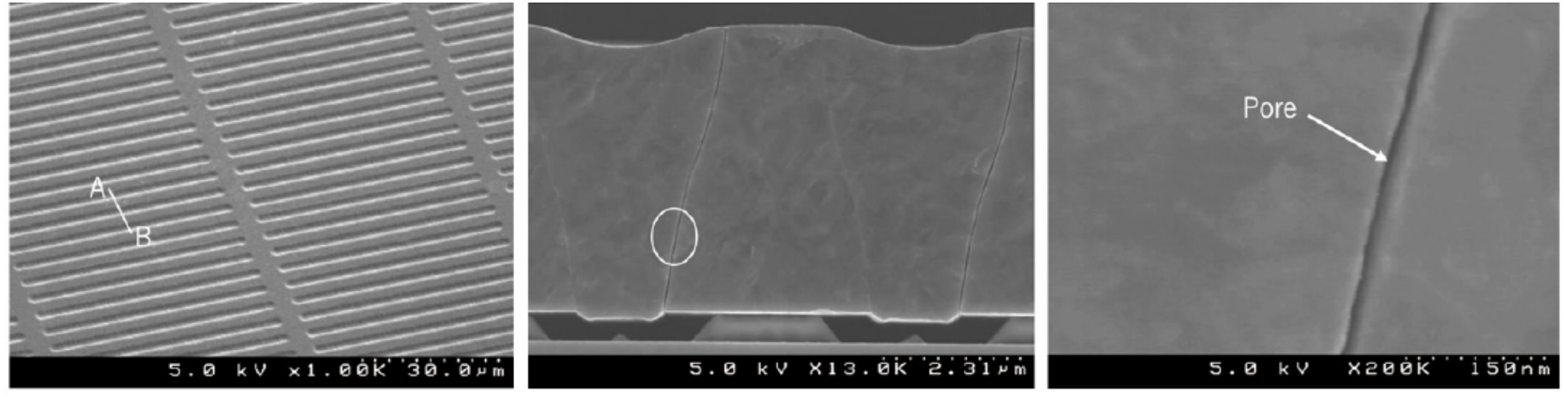
SEM Images of Standard Silicon Nanopore Membranes for hemofiltration. (Left) Top view showing the uniform array of slit pores. (Center) Cross-section image showing the non-tortuous path of the pore. (Right) A close up image of the slit pore showing the smooth surface characteristics. Reprinted from *W*. *H Fissell et al*. */ Journal of Membrane Science* under a CC BY license, with permission from Elsevier, original copyright 2009.

Our group has used MEMS technology to pioneer a novel and mechanically robust silicon nanopore membrane (SNM) for implantable renal replacement therapy. These “standard” SNM, shown in Fig 1, were designed to mimic the slit pore geometry of glomerular podocytes and enabled an order of magnitude higher hydraulic permeability over commercially available hollow-fiber membranes [14,19,20]. The uniform pore size distribution of the SNM allow for selective size based hemofiltration with strict molecular weight cut-offs, not achievable with polydisperse pores sizes of conventional polymer membranes typically used in dialyzer cartridges.

We hypothesized that the uniform pore size distribution of SNM would also result in improved selectivity and clearance for hemodialysis applications. To enhance the diffusive properties of SNM, we developed a MEMS fabrication process to reduce the effective diffusion path length of the membranes down to 100 μm. Here we present the modeling, fabrication, and in vitro and extracorporeal clearance data for the “diffusive-SNM” for use in hemodialysis applications.

## Materials and Methods

### Model Formulation

**Fig 2:**
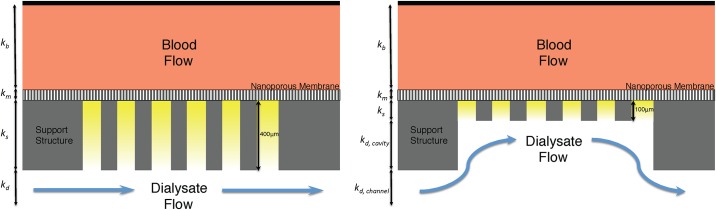
Support Structure Reduction. (Left) Schematic of the standard-SNM with a 400 μm thick support structure that results in a longer diffusion path and decreased diffusive transport. The mass transfer coefficients for blood (k_b_), membrane (k_m_), support structure (k_s_) and dialysate (k_d_) are shown in the figure. (Right) Depicts the diffusive-SNM with a reduced support structure (100 μm) resulting in improved diffusive clearance due to the decreased resistance from the support structure. The mass transfer coefficients for k_d_ is rework to take into account the cavity (k_d, cavity_) and the channel (k_d, channel_).

Fig 2 shows a schematic of the SNM at two support structure thicknesses demonstrating the change in diffusion length. The solute flux (*J*_*s*_) is a function of the difference between bulk blood (*C*_*b*_) and dialysate concentrations (*C*_*d*_), such that *J*_*s*_ = *k*_*T*_(*C*_*b*_ − *C*_*d*_), where *k*_*T*_ is the total (or overall) mass transfer coefficient. Here we assume that, in the absence of any transmembrane applied pressure and negligible solute osmotic pressure gradients, transport through SNM is exclusively a result of diffusion. The change in bulk concentrations with time for blood and dialysate compartments in a batch dialyzer is

$$\frac{dC_{b}}{dt}=-\frac{k_{T}(C_{b}-C_{d})A_{m}}{V_{b}}$$ (1)

$$\frac{dC_{d}}{dt}=\frac{k_{T}(C_{b}-C_{d})A_{m}}{V_{d}}$$ (2)

In Eqs 1 and 2, *A*_*m*_ is the membrane area while *V*_*b*_ and *V*_*d*_ are the blood and dialysate reservoir volumes, respectively. The total mass transfer coefficient can be evaluated using a resistance-in-series framework (Fig 2) and is given as

$$\frac{1}{k_{T}}=\frac{1}{k_{b}}+\frac{1}{k_{m}}+\frac{1}{k_{s}}+\frac{1}{k_{d}}$$ (3)

where the intrinsic mass transfer coefficients of the membrane and support, *k*_*m*_ and *k*_*s*_, respectively, are functions of the membrane or support thickness and the effective diffusivity of the solute in the respective layer (*D*_*eff*_) through *k* = *D*_*eff*_/*δ*, where *δ* is the membrane thickness. The effective diffusivity is itself a function of the membrane porosity (*ε*), partition coefficient (*ϕ*), solute free diffusivity (*D*_∞_), and diffusive hindrance factor (*K*_*d*_)[25],

$$D_{eff}=ED_{\infty }\phi K_{d}$$ (4)

For slit pore membranes, the partition coefficient can be evaluated as a function of the solute radius to slit pore half width ratio, *λ*, through *ϕ* = 1−*λ*. [26] This relationship only accounts for the steric contribution to partitioning, and does not consider electrostatic, acid-base, or van der Waals interactions. Likewise, the diffusive hindrance factor for slit pores can be assumed to be [27]

$$K_{d}=\frac{1+\frac{9}{16}\lambda \text{log}\lambda -1.19358\lambda +0.4285\lambda^{3}-0.3192\lambda^{4}+0.08428\lambda^{5}}{1-\lambda }.$$ (5)

Eqs 4 and 5 are rigorously valid only for hard-sphere solutes; they can be applied to other solutes by defining *λ* in terms of an effective hard sphere radius. The external mass transfer coefficients for the blood and dialysate compartments, *k*_*b*_ and *k*_*d*_, respectively, can be taken as *k* = Sh*D*_∞_/*d*_*h*_ where *Sh* is the Sherwood number and *d*_*h*_ is the hydraulic diameter of the flow channel. For a long, thin rectangular channel, the Sherwood number can be approximated by [28]

$$Sh=1.62{\left(\text{Re}Sc\frac{d_{h}}{L}\right)}^{0.33}$$ (6)

where Re = *ρu*_*b*_*d*_*h*_/*μ* is the Reynolds number, equal to the ratio of momentum forces to viscous forces, and *Sc* = *μ*/*ρD*_∞_ is the Schmidt number and is equal to the ratio of momentum diffusivity and mass diffusivity. As the SNM forms a large cavity under the membrane support abutting the main dialysate channel, the dialysate side external mass transfer coefficient must be corrected to account for a larger effective channel height. Thus, *k*_*d*_ is reworked as $k_{d}^{-1}=k_{d,channel}^{-1}+k_{d,cavity}^{-1}$ where *k*_*d*,*channel*_ and *k*_*d*,*channel*_ are the channel and cavity mass transfer coefficients, respectively. An inherent assumption in applying this approach is that the support side cavity can be modeled as a distinct rectangular channel despite opening into the larger dialysate flow channel. The error in applying this approach is likely to be small, especially in light of the relatively small contribution of the cavity and dialysate side mass transfer resistances relative to the support mass transfer resistance and the large aspect ratio of the cavity (length to depth ratio of > 10). In both cases, *ρ* is the solvent density and *μ* is the solvent viscosity, each assumed to be that for pure water at 25 degrees Celsius.

MATLAB was used to solve the system of ordinary different equations for concentration change. The initial blood-side creatinine concentration was taken as 10 mg/dl, blood-side and dialysate reservoir volumes set at 40 ml and 500 ml, respectively, blood side and dialysate flow rates at 10 ml/min, and channel dimensions at 63 mm x 9 mm x 1.5 mm (length x width x height).

### Computational Fluid Dynamics

ANSYS FLUENT software (Version 16.2, ANSYS, Canonsburg, PA) was used for computational fluid dynamics (CFD) simulations. The CFD modeling assumed steady state, laminar flow with a Newtonian fluid. The dialysate fluid density was set to 998.2 kg/m3, the viscosity was set to 1.003e-3 kg/m*s. The dialysate flow rates were specified as inlet mass flow rates and the outlet static pressure was set to gauge pressure. A 3D simulation was run. A hexahedral mesh with 779,520 elements was used for the CFD model. The backside dialysate cavity was 3 mm wide with a 300 μm cavity height. The dialysate channel height was set at 1 mm. The dialysate flow rates were evaluated at 0.1 ml/min 1 ml/min, 10 ml/min and 100 ml/min.

### Microfabrication Process

**Fig 3:**
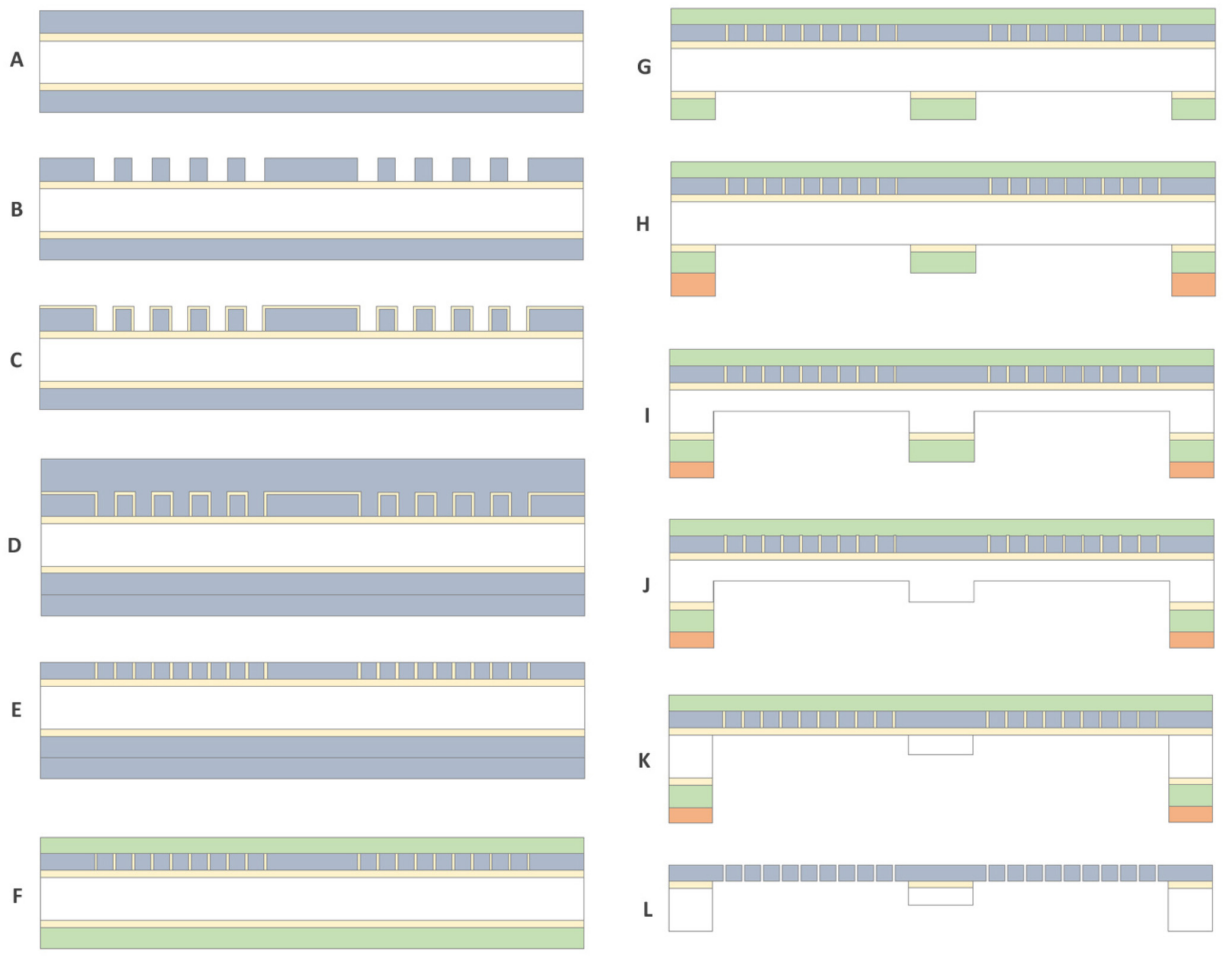
The step-wise process for the fabrication of diffusive-SNM. Using photolithography and a new two-step DRIE process that allows for reduction in the underlying support structure of the membranes. The process steps A through L are described in detail within the membrane fabrication section.

SNM were composed of a 450 nm polycrystalline silicon (polysilicon) layer with an array of precisely patterned rectangular pore slits measuring 2.3 μm x 11 nm, on a 400 μm silicon wafer serving as a mechanical support layer. Membranes were fabricated in a similar fashion with previously described microfabrication techniques [19,29]. Fig 3 shows a description of the overall fabrication process. A 500 nm thick silicon dioxide (SiO_2_) film was thermally grown on a 400 μm thick six inch double-side polished silicon substrate. Low Pressure Chemical Vapor Deposition (LPCVD) was used to deposit a 500 nm layer of undoped polysilicon at 580°C which was then annealed at 1075°C (Fig 3A) for one hour. An array of 400 nm width slits with 400 nm spacing was lithographically patterned using a stepper and etched by dry etch technique (Fig 3B). SiO_2_ was then thermally grown to 11 nm thickness at 800°C, which defines the eventual nanopores (Fig 3C). After patterning the thin SiO_2_ layer by wet etch process in buffered oxide etcher as anchors, an 800 nm thick second polysilicon layer was deposited by LPCVD (Fig 3D). The polysilicon layers were blanket-etched using dry etch until 450 nm of polysilicon remained, exposing the thin SiO_2_ on the vertical sidewall (Fig 3E) of the 400 nm slit array. After removing the polysilicon films and 11 nm SiO_2_ on the backside, 1 μm Low Temperature Oxide (LTO) was deposited by LPCVD (Fig 3F). LTO on the front side acts as a protectant layer for the remaining processes and was used for an additional oxide hard mask for DRIE on the backside. After patterning the window area on the oxide hard mask on the backside (Fig 3G), each array of windows was patterned with a 10 μm thick resist (Fig 3H).

The backside support layer was patterned with a new two-step DRIE process. The first step involves a vertical DRIE of 250 μm into the support layer within the porous region (Fig 3I). The height wall was controlled by the initial DRIE step. Following removal of the oxide hard mask (Fig 3J), a second DRIE was processed until the etched trench reaches the silicon dioxide layer over the entire porous array area resulting in a support structure thickness of 100–150 μm (Fig 3K). The variance of the support structure height comes from loading effect across the wafer during the etch process. The resulting porous array was diced into 10 mm x 65 mm devices with an effective membrane area of 2.52 cm^2^ for the in vitro experiments and 2.02 cm^2^ for the extracorporeal experiments. The device was released by dissolving silicon dioxide in HF (Fig 3L). The final process resulted in a membrane with approximately 11 nm pore widths with an effective diffusion length of 100 μm from the membrane surface to the backside dialysate surface. The surface of the membranes was inspected using scanning electron microscopy (SEM) (LEO 1550, ZEISS, Peabody, MA).

### Surface Modification

Sub-5nm thick polyethylene glycol (PEG) surface coating was performed on polysilicon surfaces [30]. Briefly, SNM were treated with a 3:1 (V/V) sulfuric acid 96% (Avantor Performance Materials, Center Valley, PA) to hydrogen peroxide 30% (Avantor Performance Materials, Center Valley, PA) solution to functionalize the polysilicon surface with hydroxyl groups. Membranes were subsequently submersed in 25 ml of toluene (Gelest Morrisville, PA), and 285 μL of PEG-silane (Gelest Morrisville, PA) at 70°C for 2 hours. Surface modified membranes were then rinsed at 10 minute intervals (3X) with toluene (Sigma Aldrich, St. Louis MO), ethanol (Decon Labs, Inc., King of Prussia, PA), and deionized water. The hydraulic permeability of SNM were determined at a pressure range between 50–300 mmHg and the average pore size was calculated using a Hagen-Poiseuille model for slit pores as cited in previous papers from our group [19,31].

### Single Channel Flow Cell

**Fig 4:**
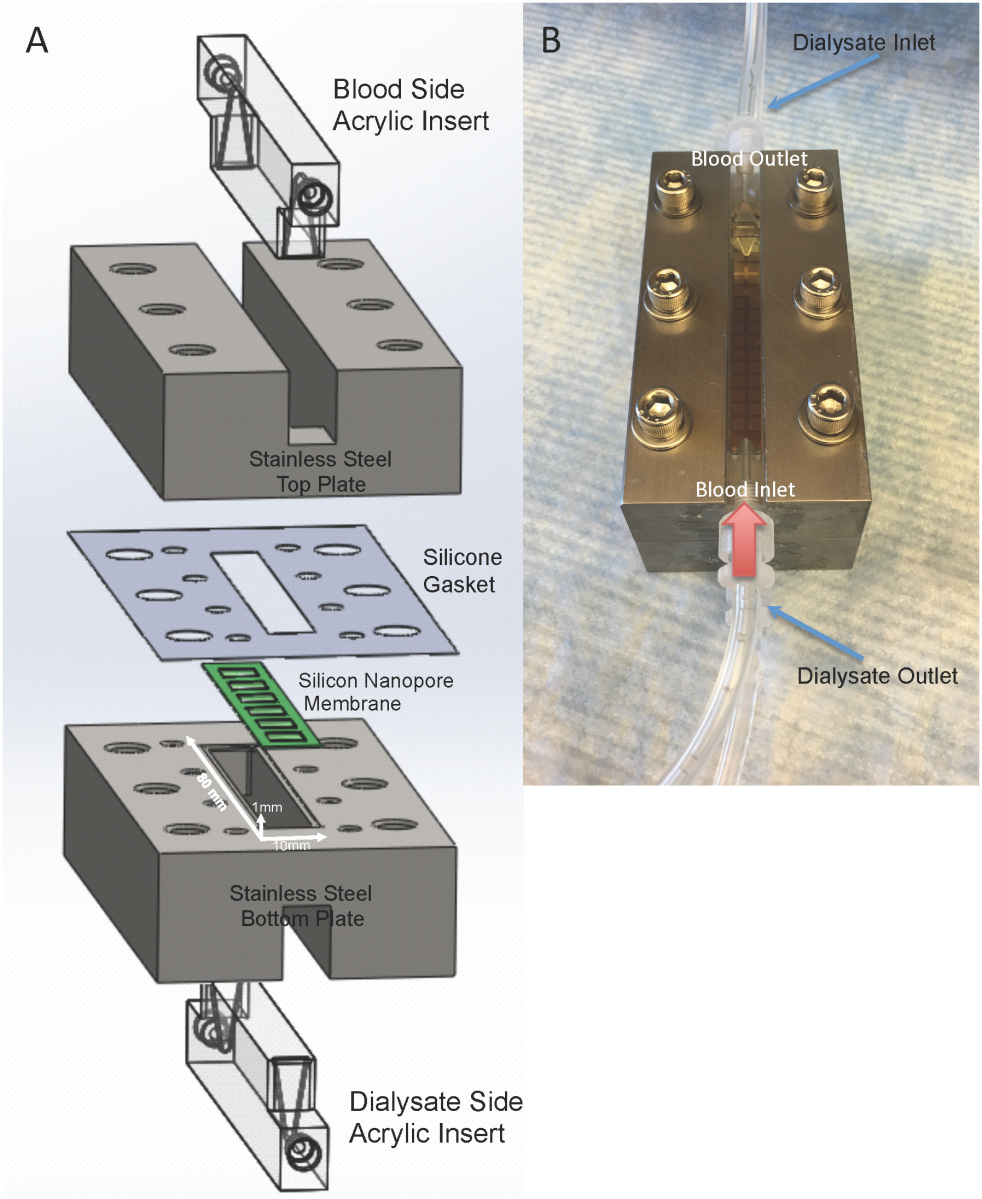
Single channel dialysate flow cell. Fig (A) shows construction of the flow cell. An acrylic insert is placed into a stainless steel plate to form the flow channels. A silicone gasket is used to form a watertight seal around the SNM. The SNM is mounted onto the bottom stainless steel plate and the dialysate side acrylic insert forms the bottom channel. The white arrows indicate the flow path dimension of 80 x 10 x 1 mm (length x width x height). The photo (B) shows the fully constructed flow cell with the blood inlet and outlet labeled on the top plate. The red arrow shows the direction of blood flow. The dialysate flows in a counter-current fashion and the blue arrow labels the dialysate inlet and outlet.

A single channel flow cell was constructed from two stainless steel plates both with an acrylic insert forming the top and bottom of the channel and allowing for visualization of the flow channel (Hayes Manufacturing, Sunnyvale, CA). Fig 4A shows a schematic of the components that are assembled to build the flow cell and Fig 4B shows a photograph of the fully assembled device. The flow path is defined by a 10 x 80 mm long channel with a height of 1 mm. A silicone gasket was used to seal the SNM and prevent fluid leaking between the two compartments. The two fluid channels had identical channel dimensions and serve as the serum and dialysate sides, respectively.

### In Vitro Diffusion Studies

**Fig 5:**
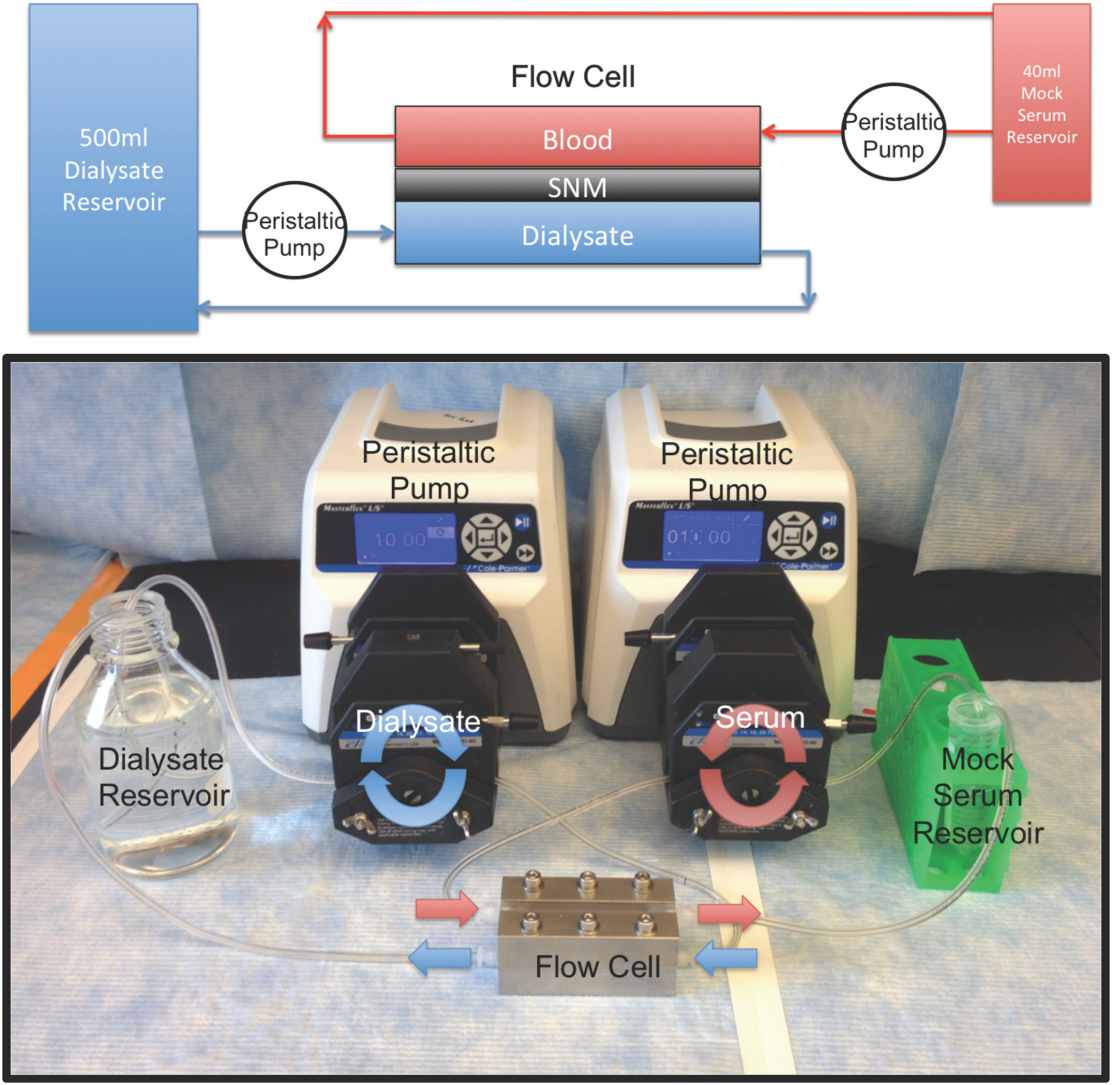
In vitro diffusion study. The top panel is a schematic of the in vitro diffusion study. Mock serum (40 ml) was pumped via a peristaltic pump over the topside of the SNM within the flow cell. The dialysate (500 ml) flows in a countercurrent direction via another peristaltic pump over the backside of the SNM. The bottom panel is a photograph of the diffusion study with the red arrows showing the direction of flow of the mock serum and the blue arrows showing the direction of flow of the dialysate.

A schematic and photograph of the in vitro diffusion study is shown in Fig 5. A 40 ml artificial serum reservoir consisting of 10 mg/dL of creatinine (Acros Organics, Geel, Belgium), 50 mg/dL of urea (Fisher Scientific, Waltham, MA), 5 mg/L of beta-2-microglobulin (Lee Biosolutions, St. Louis, MI) and 3.5 g/dL of albumin (Sigma, St. Louis, MO) in phosphate buffered saline was recirculated via a peristaltic pump at 10 ml/min (Masterflex L/S Digital Drive Peristaltic Pump, MK-07551-00, Cole Parmer, Vernon Hills, IL). Phosphate buffered saline or 0.9% sodium chloride (Baxter, Deerfield, IL) (500 ml) was used as the dialysate and flowed in a counter-current fashion at 10 ml/min via a peristaltic pump. The entire setup was passivated with the artificial serum solution for 3 hours prior to the initial time point to eliminate concentration changes due to absorption. Then samples from the artificial serum reservoir were collected hourly for 4 hours or at 0, 6, 12, and 18 hours for beta-2-microglobulin. The transmembrane pressure was maintained at zero by ensuring identical flow paths and pressures for the serum and dialysate. The volume of the serum reservoir was also closely monitored to ensure no fluid transport occurred over the course of the experiment. The concentrations of creatinine, urea and albumin were measured using an Avida 1800 Chemistry System (Siemens Medical, Erlangen, Germany) at San Francisco General Hospital (San Francisco, CA). Beta-2-microglobulin was measured via nephelometry using a SPAPlus Automated Analyzer IE610 (Bind Site Inc., San Diego, CA) at the University of California, San Francisco Clinical Laboratory (San Francisco, CA). Solute clearance (K) was calculated by fitting concentrations measured in the artificial serum at serial time points to an exponential decay function: C(t) = C_i_ *e*^*-Kt/V*^, where C(t) was the concentration at time *t*, C_i_ was the initial concentration, t was the time, and V was the volume. The dialysate solute concentration was effectively zero due to the large relative volume compared to the artificial serum reservoir.

Membrane integrity was verified by visual examination and optical microscopy followed by retesting for hydraulic permeability to determine if the average pore size had increased after the experiment. Additionally, filtrate was collected after the diffusion studies at a transmembrane pressure of 130 mmHg, with the albumin sieving coefficient evaluated as the ratio of the filtrate to feed concentrations. The initial 1 ml of filtrate was discarded to clear the internal volume of the flow cell and avoid carryover to accurately measure the filtrate concentration.

### Extracorporeal Studies

An extracorporeal blood circuit was tested for six hours in three pigs. All experiments were reviewed and approved by the University of California, San Francisco Institutional Animal Care and Use Committee. The pigs were housed in their own individual cages and had free access to food and water. The 50 kg Yorkshire pigs were given 81 mg aspirin (Bayer Healthcare, Whippany, NJ) and 75 mg clopidogrel (Bristol-Myer Squibb, Bridgewater, NJ) for 3 days prior to the procedure. The pigs were then anesthetized (Isoflurane 1–5%) and IV heparin was delivered in bolus (200 IU/kg). 15 French tunneled hemodialysis catheters (Arrow Nextstep Antegrade Chronic Hemodialysis Catheter, Teleflex Medical, Research Triangle Park, NC) were placed into the c`arotid artery and into jugular vein via cut-down and tunneled to the dorsal aspect of the pig. The pig was allowed to recover for 24 hours after placement of the catheters. The catheters were flushed and primed with 1000 units of IV heparin.

**Fig 6:**
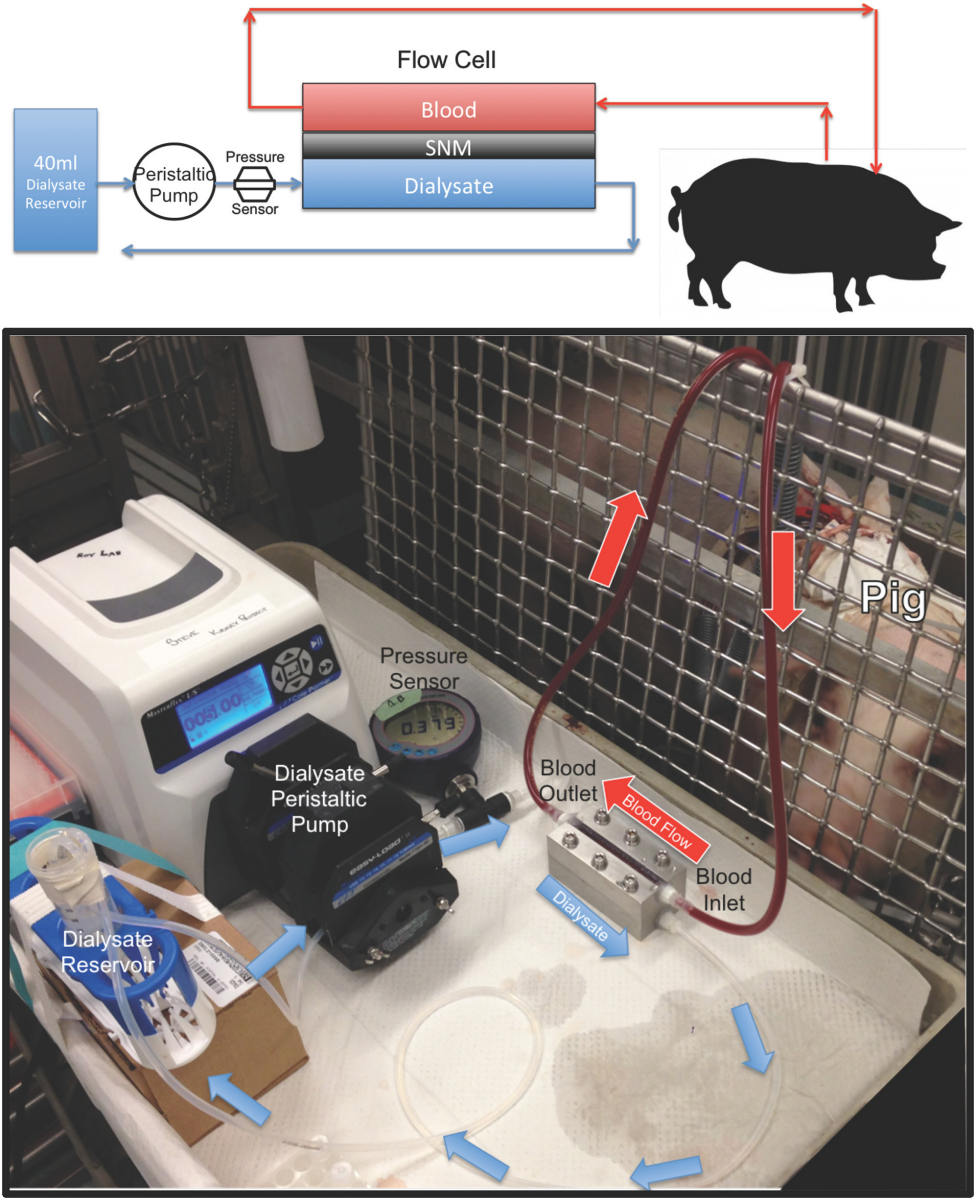
Extracorporeal diffusion study. The top panel is a figure of the extracorporeal experiment for diffusion experiments. Blood from the pig is circulated via the arterial-venous pressure differential over the top of the SNM. The dialysate flowed in a counter-current direction via a peristaltic pump over the backside of the SNM. A pressure sensor was used to measure the pressure on the dialysate side. The bottom panel shows a photograph of the extracorporeal experiment. Blood tubing was attached to the arterial catheter of the pig and blood flowed via the arterial-venous pressure differential through the flow cell. The blood was returned back to the pig via blood tubing attached to the venous catheter. The red arrows indicate the direction of blood flow. Dialysate flowed in a counter-current direction via a peristaltic pump. The blue arrows indicate the direction of dialysate flow. A pressure sensor measures the pressure on the dialysate side.

On the day of the study, the pigs were given an initial heparin bolus of 200units/kg. The flow cell was loaded with the diffusive-SNM (n = 3) and flushed with chlorhexidine (ChloroPrep, CareFusion, San Diego, CA) followed by sterile saline. The pig was placed in a crate to minimize movement during the study, which, in turn, necessitated 150 cm of tubing between the catheters and flow cell. The arterial catheter was then attached to the flow cell inlet and the outlet was connected to the venous catheter. The blood flow was then measured via timed collection at the venous outlet. The extracorporeal study is shown in Fig 6. The dialysate was 0.9% sodium chloride (Baxter, Deerfield, IL) that was recirculated in a counter-current fashion (40 ml) via a peristaltic pump at 5–7 ml/min. The flow cell was primed for 30–60 minutes prior to the start of sample collection and dialysate flow rate was adjusted to maintain zero transmembrane pressure using a pressure sensor (DPI 104 10psi, GE Druck, Leicester, UK). The timed collections were started when there was no observable change in dialysate volume over 30–60 minutes. The dialysate volume was also closely monitored throughout the experiment to ensure no ultrafiltration. Blood samples were collected from the pig at 0 and 6 hours and tested for creatinine, blood urea nitrogen, albumin, white blood cell count, hemoglobin, platelet count, lactate dehydrogenase, and C-reactive protein. The average time zero and six hour blood sample measurements were analyzed using a paired-t-test to determine if there was a significant change before and after the study. Dialysate measurements were taken hourly over 6 hours for creatinine, blood urea nitrogen and albumin. The activated clotting time was measured (iSTAT, Abbott Point of Care, Princeton, NJ) every 1.5 hours. An additional 100–200 units/kg of heparin was given as needed to maintain an active clotting time goal of > 300 seconds. The concentrations of creatinine, urea and albumin in the dialysate reservoir were measured using an Avida 1800 Chemistry System (Siemens Medical, Erlangen, Germany) at San Francisco General Hospital (San Francisco, CA). The creatinine clearance (K) was calculated from the dialysate using the equation K = (C_d_ x V) / (C_p_ x t), where C_d_ was the creatinine concentration in the dialysate, V was the volume of dialysate, C_p_ was the creatinine concentration in the plasma and t was time. The plasma concentration, C_p_, of the pig remained constant throughout the 6-hour experiment. At the conclusion of the study, the pigs were euthanized using Beuthanasia-B (sodium pentobarbital & phenytoin sodium, Merck, Madison, NJ).

At the end of the 6-hour dialysis, the flow cell was flushed with 0.9% sodium chloride (at 30 ml/min) and blood was returned back to the pig. The membranes were then examined for integrity, both visually and retested for hydraulic permeability to determine if there was an increase in the average pore size.

## Results

### Model of Diffusive Transport

**Fig 7:**
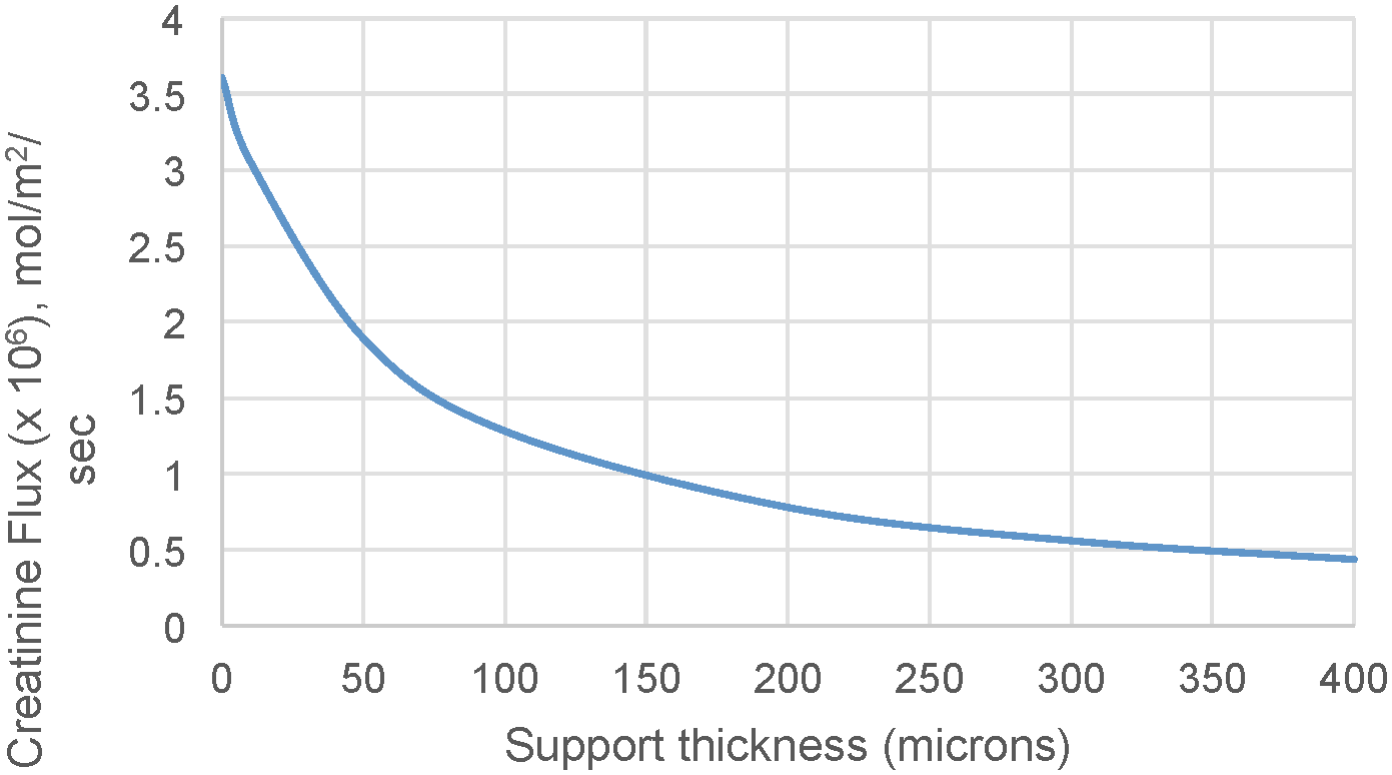
The impact of support structure thickness on creatinine flux. The graph shows the predicted change in creatinine flux across the SNM with varying support structure thicknesses.

Fig 7 shows the theoretical change in creatinine flux across a SNM with different support thickness. The model used a pore size of 10 nm and a porosity of 1%. It is clear from the modeled data that solute flux increases significantly with decreasing support thickness. The relationship between the different mass transfer coefficients (blood channel, membrane, support, backside cavity, and dialysate channel) were analyzed based on the model. For cases of zero membrane support thickness and zero dialysate side cavity thickness, there is not a corresponding mass transfer coefficient for the respective feature. The predominant source of mass transfer resistance is the membrane support, followed by blood and dialysate channel mass transfer resistance.

**Fig 8:**
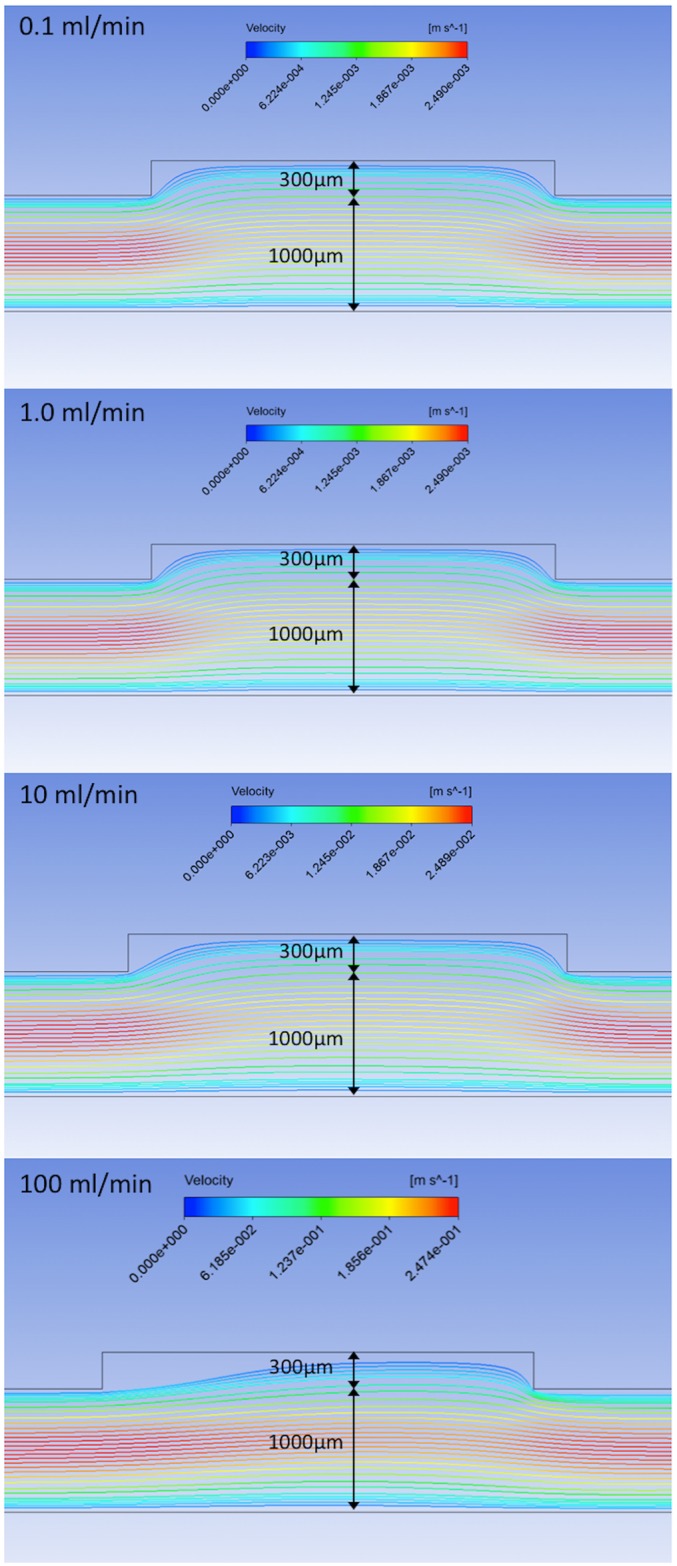
CFD Analysis of Dialysate Flow. The streamlines show the fluid velocity as it passes under the 300 μm reduced support structure of the SNM. The channel height is set to 1 mm. The various flow rates demonstrate that the flow is quickly fully developed within the cavity, except at higher flow rates (100 ml/min) when stagnant regions form under the upstream 1/3 of the backside cavity.

CFD analysis was performed to determine whether the backside cavity created by the 100 μm support structure height would result in regions of stagnant flow under the membrane that would limit diffusion rates. The CFD simulations were performed using a 300 μm backside cavity to simulate the reduction in support structure to a final SNM thickness of 100 μm. The CFD analysis is shown in Fig 8 at flow rates of 0.1, 1, 10, and 100 ml/min. The simulations demonstrate that for flow rates of 10 ml/min and below there are minimal regions of stagnant flow under the membrane. The flow becomes fully developed and laminar flow is achieved under the majority of the membrane. Thus, the backside cavity was treated in a manner similar to other rectangular channels with a characteristic external mass transfer coefficient.

### Membrane Fabrication of Diffusive Silicon Nanopore Membranes

**Fig 9:**
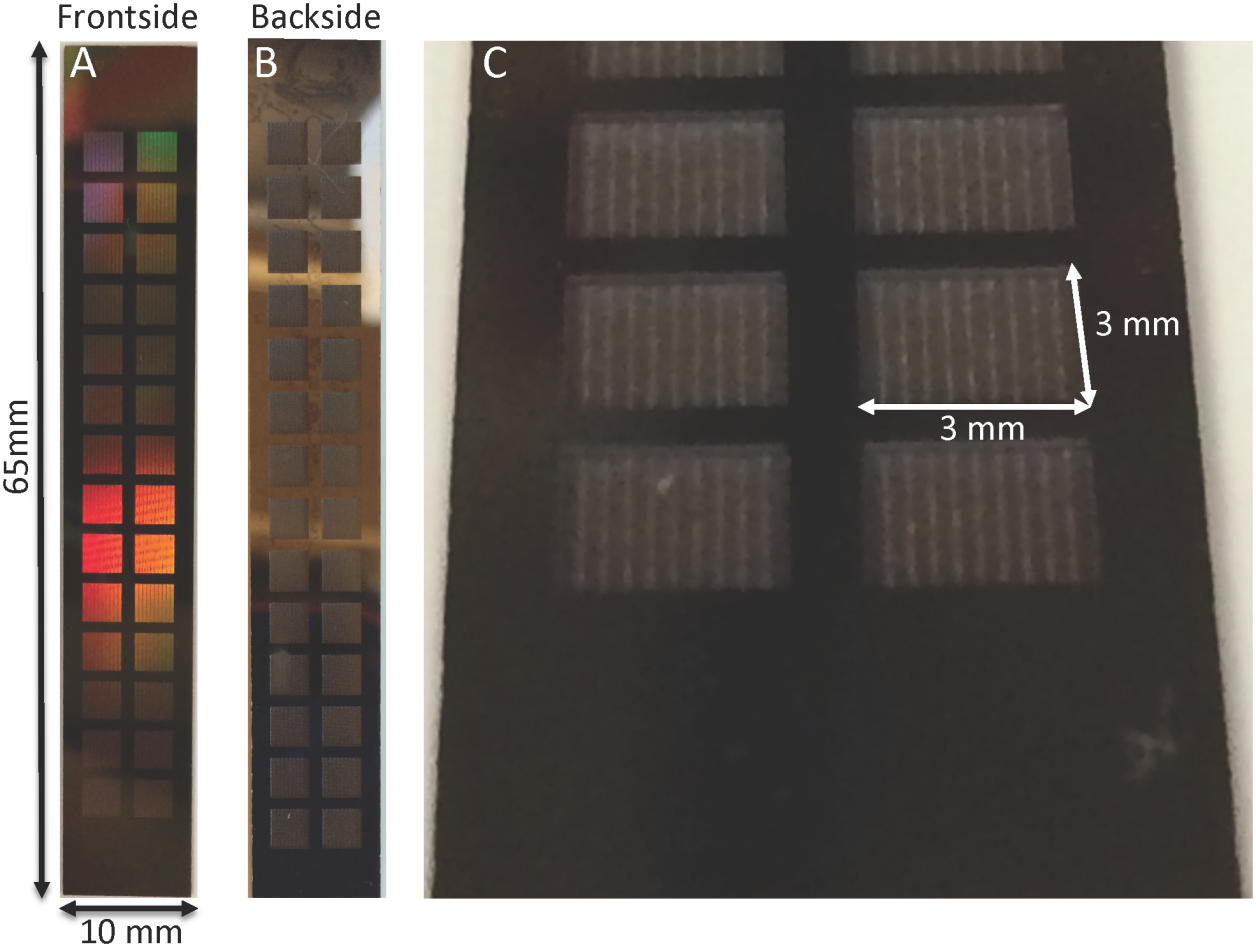
Photographs of diffusive-SNM. (A) Shows the blood-contacting surface (front side) of the SNM. The total dimensions of the SNM are 65 x 10 mm. The functioning membrane portions with pores are the 3 mm x 3 mm squares arranged within the chip. The effective membrane surface area is 2.52 cm^2^. The remaining regions are solid silicon support. (B) The dialysate-contacting surface (backside) of the SNM. (C) Close-up of the 3 mm x 3 mm backside and the recessed cavity formed by the new two-step DRIE process.

A novel microfabrication process was developed (Fig 3) based on the model results, which indicated that significant improvement in diffusion could be achieved by reducing the support structure to 100 μm. Diffusive-SNM were successfully fabricated using the new two-step Deep Reactive Ion Etching (DRIE) process that resulted in an overall reduction in the support structure height by 300 μm. A prototype of the diffusive-SNM is shown in Fig 9 and it has a total effective membrane surface area of 2.52 cm^2^. Fig 9C shows a close up of the recessed backside cavity that results in the reduced support structure of the membrane. The SNM were able to maintain mechanical integrity at pressures up to 300 mmHg during hydraulic permeability testing.

**Fig 10:**
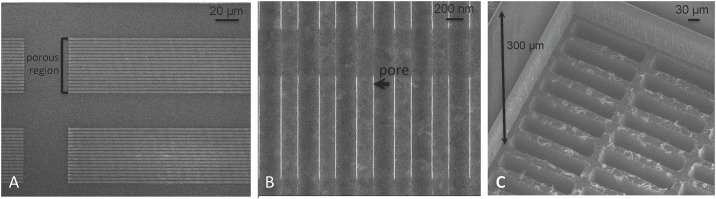
Scanning electron microscopy. (A) Depicts the porous region within a 3 mm x 3 mm portion of the SNM. Each 3 mm x 3 mm has a total of 370 porous regions that are composed of slit-pores. (B) Depicts a pore (black arrow) within a porous region. Each porous region contains ~11,000 pores. The total number of pores on an SNM is 1.14 x 10^8^. (C) Depicts the backside of the SNM and demonstrates the 300 μm reduced support structure after the two-step DRIE process.

The thickness of the support structure was measured with confocal microscopy (LEXT OLS4000, Olympus, Tokyo, Japan) and ranged between 100–150 μm. Scanning electron microscopy was used to visualize the membrane, with typical images shown in Fig 10. The image 10B shows the uniform distribution of slit-pores of the SNM. Image 10C shows the backside of the SNM after the two-step DRIE process resulting in removal of 300 μm of support structure which creates the backside cavity.

### In Vitro Diffusion Studies

Hydraulic permeability testing showed a calculated average pores size of 8.2 ± 2.0 nm (91.0 ± 67.8 ml / hr / mmHg / m^2^) after PEG coating for the SNM used in the in vitro studies. The measured pressure drop across the flow cell was 5 mmHg at 10 ml/min flow rate. A 40 ml artificial serum reservoir containing creatinine, urea, beta-2-microglobulin, phosphate and albumin was used to measure diffusive transport of these key solutes. The dialysate volume was 500 ml and was recirculated at 10 ml/min. The serum concentration drop of select solutes was undetectable using single pass measurements at 10 ml/min, indicating that the diffusion of solutes was independent of flow rate. Therefore, serum was recirculated at 10 ml/min and the solute drop followed an exponential decay function characteristic of diffusive transport. There was no volume change in either the serum or dialysate reservoir and transmembrane pressures remained zero throughout the study. The calculated clearance values for creatinine (n = 7), urea (n = 8), phosphorus (n = 5) and beta-2-microglobulin (n = 5) are shown in Table 1. Membrane integrity was verified by visual examination followed by retesting for hydraulic permeability to ensure that there were no significant changes in pore size or loss of membrane integrity after the experiment. The average pore size change before and after experimentation was -1.7 ± 2.2 nm, which is statistically insignificant; the small negative value could be due to low levels of protein adsorption within the pores. There was no significant albumin transport observed. The average change in albumin concentration was 1.2 ± 1.3% for the course of the experiments and average albumin sieving was 0.025 ± 0.03. The expected coefficient of variation for albumin using the Avida 1800 was 2.1–2.3% based on the June 2015 Quality Report at the San Francisco General Hospital Clinical Laboratory.

**Table 1:** In vitro Clearance values normalized to surface area (ml/min/m^2^).

	Creatinine	Urea	Phosphorus	Beta-2-microglobulin
**Diffusive-SNM**	109 ± 14	150 ± 21	87 ± 13	27 ± 8
**Standard-SNM**	53 ± 7	63 ± 4	45 ± 3	13 ± 4
**Improvement**	2.1X	2.4X	1.9X	2.1X

The values are means ± standard deviation. The clearance values are normalized for surface area (2.52 cm^2^).

**Fig 11:**
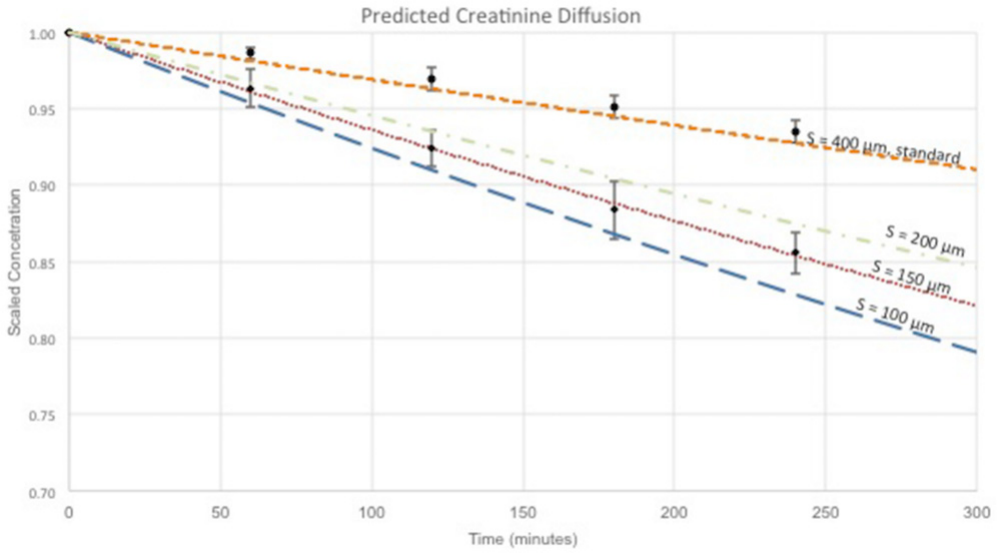
Creatinine Transport Model of SNM. The graph shows the scaled concentration decrease over time for creatinine via diffusion only. The colored lines represent the predicted concentration over time at various support structure heights (400, 200, 150 and 100 μm) based on the mathematical model for diffusive-SNM and standard-SNM. The black circles represent experimental data obtained in vitro for standard-SNM design with 400 μm support structure. The black diamonds represent experimental data obtained in vitro for the diffusive-SNM design with support structures between 100–150 μm.

The results of the predicted creatinine transport with support structure heights of 100, 150, and 200 μm and the standard-SNM (structure height of 400 μm) are shown in Fig 11. Note that the pore structure of the diffusive-SNM is slightly different than the standard SNM, with the former possessing higher overall porosity. The reduction of the support structure from 400 μm down to 100 μm results in a 2.3-fold increase in creatinine clearance. The experimental results for creatinine diffusion over time are also plotted in Fig 11 for the diffusive-SNM and standard-SNM, with the data in good agreement with the diffusive transport predicted by the model.

### Extracorporeal Studies

Diffusive transport of creatinine was tested in an extracorporeal blood circuit for six hours in three pigs. The flow cell was loaded with the SNM (n = 3) and the pore size for the SNM used in the extracorporeal studies was 7.9 ± 0.3 nm. The flow cell inlet was connected to the arterial catheter and the outlet was connected to the venous catheter. The blood flow was achieved via the arterial venous pressure differential and ranged between 35–120 ml/min depending on catheter position. The average blood flow was 93 ± 36 ml/min. The extracorporeal circuit was run for 6 hours and the membranes were then examined for integrity, both visually and retesting for hydraulic permeability to verify that there was not a significant increase in pore size. The average pore size change following blood exposure was -1.9 ± 1.2 nm.

There was one episode of arterial catheter clot that resulted in cessation of flow. The flow cell was disconnected and the catheter was aspirated and flushed with 0.9% sodium chloride. Blood flow was reestablished after flushing the catheter and the flow cell was reconnected. There was no evidence of visible thrombus on the SNM upon completion of the 6-hour study for any of the experiments.

The calculated creatinine clearance value normalized to surface area was 85 ± 18 mL/min/m^2^. The albumin concentration in the dialysate was zero in all of the dialysate reservoirs after 6 hours. The serum urea concentration of the pigs was less than 10 mg/dl and the urea concentration in the dialysate ranged between 1–3 mg/dL after 6 hours. The precision of the assay was too low to accurately calculate a diffusive clearance for urea given the small concentration gradient between the blood and dialysate. The changes in phosphorus and beta-2-microglobulin concentrations were also too small to accurately calculate a diffusive clearance given the sensitivity of our tests.

The pigs tolerated the 6-hour procedure and did not have clinical deterioration or adverse effects. The blood samples taken at zero and six hours did not show a significant change (p < 0.05) for any values measured, except for the platelet count which increased by more than a factor of two after 6 hours for the diffusive-SNM (S1 Table).

## Discussion

Our laboratory has previously described and characterized the standard-SNM with unprecedented hydraulic permeability for use as a hemofilter in a fully implantable bioartificial kidney [19]. The uniform pore size distribution allows for discrete size based filtration, which would also be advantageous for diffusion based therapies. However, the diffusive clearance of solutes was hindered by the thick support structure (400 μm) of the SNM. The model for diffusive transport of solutes through a SNM with the support structure reduced down to 100 μm predicted an improved diffusive clearance of 2.3-fold. Additionally, CFD simulations showed that the reduced dialysate side support structure still allows for dialysate fluid to enter the backside cavity and fully develop laminar flow at the interface at flow rates of 10 ml/min and below. Therefore, the concentration of solutes at the surface of the 100 μm support structure is the same as the bulk concentration, effectively zero. The significant contribution of the membrane support in limiting solute flux motivated the development of a novel microfabrication process of the diffusive-SNM.

The fabrication process detailed here significantly reduce the overall thickness of the silicon support for the SNM to 100–150 μm. The diffusive-SNM were able to maintain mechanical integrity up to 300 mmHg in vitro without fracture, which is higher than any transmembrane pressure encountered clinically. The diffusive-SNM were able to maintain equivalent hydraulic permeability as the standard-SNM. However, we observed a 2.1-fold improvement in creatinine clearance with the diffusive-SNM compared to the standard-SNM in both in vitro and extracorporeal studies. Based on the predicted model, there is a 2.3-fold improvement by reducing the support structure down to 100 μm. The experimental results for creatinine clearance were consistent with the model calculations assuming a support structure height between 100 and 150 μm (Fig 11). The measured support structure height based on confocal imaging also ranged between 100–150 μm. The results of the model closely approximate the experimental results, with the small difference between the model and experimental results likely due to membrane fouling and gasket creep onto the membrane area, both of which were not accounted for in the model. The discrepancy in the modeled data for diffusive-SNM and standard-SNM at 400 μm is explained by the slightly different pore arrangements for the two membrane types. The diffusive-SNM would, therefore, be able to perform clearance in both diffusive and convective modes.

The beta-2-microglobulin clearance of the diffusive-SNM was 18% of the urea clearance. Current high flux hemodialyzers have beta-2-microglobulin clearances of about 5–8% of urea clearance [32,33]. Additionally, beta-2-microglobulin has significant absorption to polymer membranes which accounts for 55% of beta-2-microglobulin removal with polysulfone and >95% for polyacrylonitrile and polyethyleneimine membranes [34]. The beta-2-microglobulin results presented here were conducted carefully to ensure there was no significant absorption during the experiments. The membranes and flow circuit were passivated with high concentrations of albumin (3 g/dL) for 3 hours prior to testing. Additionally, we performed transport studies with solid silicon membranes to show that the beta-2-microglobulin concentration did not change over 24 hours following passivation. This passivation step ensured that the change in beta-2-microglobulin concentration was due to diffusion across the membrane and not absorption. Additionally, albumin sieving was performed after the experiment to ensure that membrane integrity remained intact and transport was not through cracks. The improved beta-2-microglobulin transport of the SNM is likely due to the uniform slit-pore distribution of the SNM. The precise slit-pore geometry allows for better middle molecule clearance of the SNM over current commercial hollow-fiber membranes with larger pore size distributions.

The in vitro urea clearance of the diffusive-SNM was 150 ± 21 ml/min/m^2^. The single pass measurements at a serum and dialysate flow rate of 10 ml/min did not show a change in concentration, and thus, we can assume the transport is no longer dependent upon flow rates. Current high-flux dialyzers have urea clearances normalized to surface area of 130 ml/min/m^2^ at blood flow rates of 200 ml/min and dialysate flow rates of 500 ml/min and clearances of 230 ml/min/m^2^ at higher flow rates of 400 ml/min and dialysate flow at 800 ml/min [33]. These clearance values demonstrate that at clinically relevant flow rates the diffusive-SNM are comparable to current commercial hemodialyzers. Obviously, given that commercial polymer membranes have thicknesses ranges of 35–40 μm the mass transfer coefficient—area (K_o_A) values are typically between 560–760 ml/min/m^2^ [35]. However, this is only a theoretical clearance given that the safest maximum blood flow rate is around 450 ml/min due to the vascular access. Furthermore, the clearance of urea and other small, extra-cellular, water soluble molecules maybe less important as a benchmark for frequent or intensive hemodialysis as these molecules are rather quickly cleared after a few hours. The use of urea clearance as a measure of dialysis adequacy is more relevant for conventional, three hour, thrice weekly hemodialysis where large amounts of small solutes need to be quickly removed. For frequent or intensive hemodialysis, middle molecule clearance is likely to be a more relevant benchmark.

The extracorporeal porcine studies showed that blood could successfully be driven through a parallel plate channel without the aid of a blood pump. This is obviously not a novel finding and has been used with parallel plate dialyzers in the past, but it is a conceptual shift away from practices with current hollow-fiber membranes [36]. We successfully operated the extracorporeal device over 6 hours without complete blood circuit thrombosis or clinical deterioration of the pig. There was a 22% reduction in creatinine clearance by the SNM between the in vitro studies and the extracorporeal studies; this reduction is likely due to concentration polarization and fouling from the blood environment. The SNM are coated with a thin-film polymer (PEG) to prevent biofouling and thrombosis [14,19], while still maintaining pore patency. Our SNM allow for improved hemocompatibility via thin-film coatings, thereby enabling the potential for long-term operation not feasible with current polymer membranes [19,30], but further work needs to be performed to improve overall hemocompatibility of the SNM.

The significant improvement in diffusive clearance with the diffusive-SNM could allow for use in new systems for frequent or intensive hemodialysis. The recommended minimum single pooled Kt/V to achieve a standard Kt/V of 2.0 per week is 0.34 for an 8 hour dialysis session, 7 days a week. This target would require a clearance of 24.8 ml/min per treatment (for a patient with urea volume of distribution of V = 35 L) with approximately 0.17 m^2^ of SNM surface area based on the urea clearance data presented here. This clearance target would result in a device with ~20 blood channels made up of 4 x 10 cm SNM area forming both the top and bottom of the blood channel. The final device would have an internal volume of ~100 ml. An implantable or wearable device providing continuous renal replacement would need even less surface area given that peritoneal dialysis provides an average creatinine clearance normalized to 1.73 m^2^ of body surface area of 5.8–3.8 ml/min (38.3–58.6 liters weekly) [37]. This lower clearance requirement would result in a significantly more compact device with less surface area.

In this study, we presented results using membranes with a surface area of 2.52 cm^2^. Our next step is to develop larger SNM with increased surface area. We are currently in the process of microfabricating the next generation of silicon membranes with increased surface area so that we may ultimately achieve our clinical targets. Additionally, the promising results shown here demonstrate that continued optimization of the SNM design, by further decreasing the support structure and increasing overall porosity, could result in an additional 2-fold increase in clearance. This enhancement would result in clearance values comparable to the K_o_A values of current high flux dialyzers.

## Conclusion

Here we present data showing that diffusive-SNM with thinner support structures provide over 2-fold improved clearances compared to standard-SNM with superior relative beta-2-microglobulin clearance compared to polymer high-flux dialyzers. These results were consistent with mathematical models. We additionally demonstrated that the same degree of improvement in clearance was maintained in an extracorporeal porcine study The SNM show early promise as a compact, highly efficient hemodialysis membrane for use in a portable or extended use device. This SNM based device could offer an alternative to current traditional dialysis modalities with important clinical and quality of life benefits for patients. However, significant work will be needed to improve transport and address long-term blood contacting issues with the SNM in order for clinical feasibility to be achieved. As a next step, we will pursue animal studies to evaluate the long-term feasibility of SNM for continuous or extended renal replacement therapy followed by testing in an ESRD model.

## Supporting Information

S1 Table Extracorporeal Porcine Laboratory Results: Pre and Post Experiment. The values are means ± standard deviation (n = 3). The 6-hour diffusive-SNM showed a significant increase in platelet concentration (p < 0.05). All other values did not show a significant difference.
(DOCX)
