# Supplementary material for: Diffusive Silicon Nanopore Membranes for Hemodialysis Applications
Source: PLoS One. 2016 Jul 20;11(7):e0159526. doi: 10.1371/journal.pone.0159526 (PMC4954641; doi:10.1371/journal.pone.0159526)
Supplement: S1 Table — The values are means ± standard deviation (n = 3). The 6-hour diffusive-SNM showed a significant increase in platelet concentration (p < 0.05). All other values did not show a significant difference. (DOCX) [file pone.0159526.s001.docx]

**S1 Table** Extracorporeal Porcine Laboratory Results: Pre and Post Experiment

| Standard-SNM | Pre (t = 0hr) | Post (t = 6hr) | p-value |
| --- | --- | --- | --- |
| Creatinine (mg/dL) | 1.45 ± 0.07 | 1.45 ± 0.10 | 0.92 |
| Blood Urea Nitrogen (mg/dL) | 11 ± 2 | 11 ± 2 | 0.44 |
| Albumin (g/dL) | 3.3 ± 0.2 | 3.3 ± 0.2 | 1.0 |
| White blood cell count (k/ul) | 14.6 ± 4.5 | 19.7 ± 8.4 | 0.15 |
| Hemoglobin (g/dL) | 9.4 ± 1.1 | 9.5 ± 1.6 | 0.77 |
| Platelets (k/ul) | 153.3 ± 67.6 | 206.7 ± 64.7 | 0.42 |
| C-reactive Protein (mg/L) | 1.0 ± 0.1 | 1.2 ± 0.2 | 0.12 |
| Lactate Dehydrogenase (U/L) | 892 ± 214 | 853 ± 216 | 0.40 |

| Diffusive-SNM | Pre (t = 0hr) | Post (t = 6hr) | p-value |
| --- | --- | --- | --- |
| Creatinine (mg/dL) | 1.29 ± 0.02 | 1.31 ± 0.10 | 0.78 |
| Blood Urea Nitrogen (mg/dL) | 9 ± 2 | 10 ± 2 | 0.74 |
| Albumin (g/dL) | 3.1 ± 0.3 | 3.3 ± 0.1 | 0.19 |
| White blood cell count (k/ul) | 17.1 ± 5.7 | 22.7 ± 4.5 | 0.46 |
| Hemoglobin (g/dL) | 10.2 ± 1.2 | 10.6 ± 1.4 | 0.12 |
| Platelets (k/ul) | 134.3 ± 48.8 | 327.7 ± 31.0 | *0.012* |
| C-reactive Protein (mg/L) | 1.33 ± 0.95 | 1.7 ± 1.1 | 0.09 |
| Lactate Dehydrogenase (U/L) | 1214 ± 307 | 1041 ± 422 | 0.16 |

The values are means ± standard deviation (n=3). The 6-hour diffusive-SNM showed a significant increase in platelet concentration (p < 0.05). All other values did not show a significant difference.
